# The agreement of low lean mass with obesity using different definitions and its correlation with hyperuricemia

**DOI:** 10.3389/fnut.2024.1382254

**Published:** 2024-04-02

**Authors:** Huan Xiao, Longxiangfeng Hu, Mengyu Xie, Yunfei Du, Dan Liao

**Affiliations:** ^1^Department of Radiology, Mianyang Central Hospital, School of Medicine, University of Electronic Science and Technology of China, Mianyang, China; ^2^Chengdu Medical College, Chengdu, China; ^3^Department of Nephrology, Mianyang Central Hospital, School of Medicine, University of Electronic Science and Technology of China, Mianyang, China

**Keywords:** hyperuricemia, low lean mass, National Health and Nutrition Examination Survey, obesity, sarcopenia, sarcopenic obesity

## Abstract

**Background:**

The agreement on the identification of sarcopenic obesity remains elusive, and its association with hyperuricemia remains unestablished. This study sought to evaluate the agreement of low lean mass (LLM) with obesity and its correlation with hyperuricemia.

**Methods:**

A total of 25,252 study participants, comprising 4,597 individuals with hyperuricemia, were obtained from the National Health and Nutrition Examination Survey spanning the years 1999–2006 and 2011–2018. LLM with obesity was characterized by the coexistence of LLM, determined by the ratio of appendicular lean mass to body mass index (BMI), and three categories of obesity including BMI, body fat percentage (BF%), and waist circumference (WC). We employed Cohen’s kappa to evaluate the agreement among the different diagnostic criteria and implemented survey multiple logistic regression and stratified analyses to explicate the connection between LLM with obesity and the risk of hyperuricemia.

**Results:**

When defining obesity using BF%, BMI, and WC, the prevalence of LLM with obesity varied from 6.6 to 10.1%, with moderate-to-strong agreement. In the fully adjusted model, individuals with LLM or any of the three types of obesity exhibited notably elevated odds of developing hyperuricemia. Likewise, participants with LLM and obesity had 2.70 (LLM + BMI), 2.44 (LLM + BF%), and 3.12 (LLM + WC) times the risk of hyperuricemia, respectively, compared with healthy individuals. The association between LLM with obesity and hyperuricemia remained stable and significant across different age and sex subgroups.

**Conclusion:**

When employing the three definitions of obesity, the incidence of LLM with obesity was not high, and the diagnostic agreement was relatively good. The participants with LLM and obesity exhibited an increased risk of hyperuricemia.

## Introduction

Hyperuricemia, a burgeoning health concern with implications for individuals of diverse age groups and genders worldwide, arises primarily from dysfunctions in purine metabolism and uric acid excretion (1). The occurrence of hyperuricemia has risen globally particularly in developed countries because of the shift in lifestyle and diet patterns. It was reported that the global incidence of hyperuricemia fluctuates between 9.3 and 20.1% and is accompanied by a trend of younger (2–5). Hyperuricemia is widely recognized as a contributing factor to gout and is also associated with an unfavorable prognosis primarily due to the elevated risk of complications, particularly chronic kidney disease, diabetes, hypertension, and cardiovascular disease (CVD) (6–9). Consequently, identifying modifiable factors is critical for preventing hyperuricemia.

Sarcopenic obesity (SO) is a condition characterized by a decline in muscle mass (sarcopenia) accompanied by excessive adipose tissue (obesity) (10). Muscle loss in obese subjects is not only strongly related to an increased likelihood of abnormal lipid metabolism, hypertension (11), diabetes (12), and CVD (13) but is also remarkably linked to an elevated risk of mortality (14, 15). Obesity has been demonstrated to be linked to hyperuricemia, where obesity is defined as body mass index (BMI), weight-adjusted-waist index (WWI), total percentage fat, or waist circumference (WC) (16–18). Previous investigation demonstrated a negative association between grip strength and serum uric acid in individuals aged 20–40 years. However, this association appears to be reversed in individuals aged 60 and above (19). Sarcopenia and obesity may have a synergistic effects, leading to adverse health problems.

The association between low lean mass (LLM) with obesity and hyperuricemia remains uninvestigated in the existing literature, with further research needed to elucidate potential variations across diverse age groups and genders. We first aimed to assess the correlation between LLM accompanied by obesity and hyperuricemia in young (20–40), middle-aged (40–60), and elderly (≥60) individuals based on the National Health and Nutrition Examination Survey (NHANES). Considering that hyperuricemia displayed a younger trend and the incidence of sarcopenia and obesity varied across different ages and genders, we further investigated this association in different age and gender subgroups. Additionally, previous research has suggested that the incidence of SO varies extensively due to diverse obesity indicators (14, 20, 21). Consequently, we further evaluated the agreement of LLM with obesity using three different definitions of obesity in the study population. We hypothesized that individuals with LLM accompanied by obesity were associated with a higher risk of hyperuricemia.

## Methods

### Study population

The NHANES is an extensively representative health survey conducted by the National Center for Health Statistics (NCHS) employing intricate, multistage sampling techniques.

**Figure 1:**
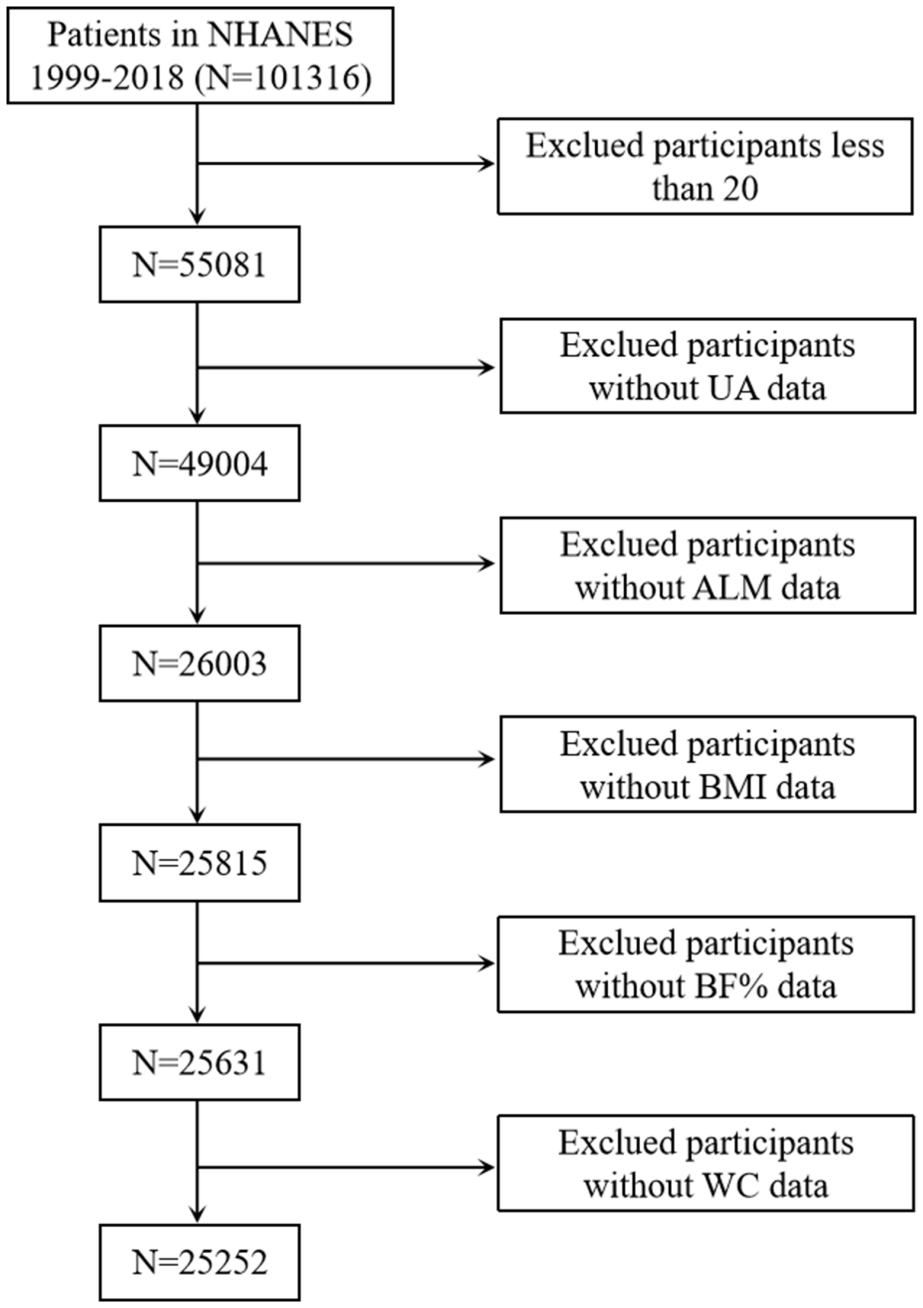
Selection of the study population. UA, uric acid; ALM, appendicular lean mass; BMI, body mass index; BF%, body fat percentage; WC, waist circumference.

Survey data spanning from 1999 to 2006 and 2011 to 2018 were selected because these period covered different body components measured using dual-energy X-ray absorptiometry (DXA). Initially, a screening cohort consisting of 101,316 individuals was enrolled. Finally, 25,252 participants were chosen after excluding individuals under the age of 20 years, those lacking body composition measurements [appendicular lean mass (ALM), BMI, body fat percentage (BF%), and WC], and those without serum uric acid information (Figure 1).

### Assessment of hyperuricemia

Hyperuricemia was characterized as having a serum uric acid level that surpassed 7.0 mg/dL in males and 6.0 mg/dL in females (8).

### Measurement of body composition

Following standard procedures and using appropriate equipment, trained health technicians measured the weight, height, and WC of the participants. BMI was calculated as weight (kg) divided by height (m) squared. ALM and BF% were obtained using DXA. ALM was determined by aggregating the muscle mass of the four limbs while excluding the body mineral content. LLM was characterized as ALM/BMI according to the criteria established by the Foundation for the National Institutes of Health (FNIH), with values below 0.789 for males and below 0.512 for females (22). The definition of obesity in this analysis was based on three distinct criteria: BMI ≥ 30 kg/m^2^ (23), BF% ≥ 30% in males and ≥40% in females (24), or WC ≥ 102 cm in males and ≥88 cm in females (25). Subsequently, the participants were classified into four distinct groups: individuals without obesity or LLM (normal), those with only LLM, those with only obesity, and those with both LLM and obesity. As a result of the existence of three distinct definitions of obesity, three alternative definitions for LLM accompanied by obesity were identified: LLM plus BMI, LLM plus BF%, and LLM plus WC.

### Covariates

The confounding factors considered in this study were as follows: sociodemographic information [age, sex, race (Black, White, Mexican, and others), educational level (less than high school, high school graduates, more than high school), poverty income ratio (PIR), smoking status (never, former, and current), alcohol (no, mild, moderate, heavy, and former), and physical activity] and comorbid medical conditions (hypertension, diabetes, and hyperlipidemia), 24 h dietary intake (total protein and energy), and laboratory metrics (blood urea nitrogen and creatinine). PIR was defined as the ratio of total family income to the poverty threshold and divided into three groups (<1.3, 1.3–3.5, and >3.5) (26). Physical activity was classified into three categories based on the American Heart Association: never, moderate, and vigorous (27). The diagnosis of diabetes met one of four different criteria: self-reported diagnosis, using diabetes medications, hemoglobin A1c ≥ 6.5%, or fasting plasma glucose ≥126 mg/dL. The definition of hyperlipidemia was based on three distinct criteria: (1) the utilization of lipid-lowering drugs; (2) triglyceride ≥150 mg/dL; (3) total cholesterol ≥200 mg/dL, high-density lipoprotein cholesterol <40 mg/dL for males or <50 mg/dL for females, or low-density lipoprotein cholesterol ≥130 mg/dL. Hypertension was determined using the utilization of antihypertension medications or systolic/diastolic blood pressure ≥140/90 mmHg.

### Statistical analysis

Following the NHANES analysis guidelines, we applied sampling weights of the population sampling examination (wtmec2yr for 2003 to 2006 and 2011 to 2018 and wtmec4yr for 1999–2002) and masked variance in R 4.2.2 to account for the intricate study design of NHANES. 8-year MEC weights were calculated as wtmec2yr divided by 8 or wtmec4yr divided by 4 (28). We compared the baseline characteristics of different body compositions by using one-way ANOVA for continuous variables or the chi-square test for categorical variables, according to three specific criteria of LLM combined with obesity. To assess the agreement between the three distinct LLM with obesity, an initial estimation was conducted to determine the prevalence of normal, LLM, obesity, and LLM accompanied by obesity. Subsequently, Venn diagrams were employed to investigate the agreement among various diagnostic criteria. Additionally, Cohen’s kappa in MedCalc version 19.1 was employed to assess the agreement between each pair of diagnostic criteria. Weighted multivariate logistic regression analysis was adopted to examine the association between body composition phenotypes and hyperuricemia across three different models. Model I was adjusted for age, sex, and ethnicity. Model II incorporated additional sociodemographic characteristics, whereas Model III added comorbid medical conditions, dietary intake, and laboratory metrics. To account for variability in body composition across various age and sex subgroups, we implemented interaction and stratified analyses based on age (20–40, 40–60, and ≥ 60 years) and sex. Due to the possible differences in this relationship between males and females across various age groups, we further conducted subgroup analyses on the youth, middle-aged, and elderly populations of different genders. Statistical significance was set at *p* < 0.05.

## Results

Among the 25,252 study participants, 4,597 subjects were identified as having hyperuricemia. These individuals had an average age of 40.45 [standard error (*SE*) = 1.00] years and a male proportion of 49.84% (*SE* = 0.01). Most participants were identified as White, accounting for 67.85% of the sample. Table 1 indicates the baseline characteristics of the three different LLM accompanied by obesity criteria. When BF%, WC, and BMI were used to classify obesity, subjects with both LLM and obesity showed the highest prevalence of hypertension, diabetes, and hyperlipidemia. Furthermore, in comparison to the normal group, LLM in obese subjects demonstrated a greater likelihood of being older and inactive. They also displayed lower levels of educational achievement, family income, and reduced protein and energy intake.

**Table 1:** General characteristics of included participants.

	LLM + BMI				*P*	LLM + BF%				*P*	LLM + WC				*P*
	Normal	LLM	Obesity	LLM with obesity		Normal	LLM	Obesity	LLM with obesity		Normal	LLM	Obesity	LLM with obesity	
	*N* = 15,313	*N* = 1,289	*N* = 6,968	*N* = 1,682		*N* = 13,721	*N* = 409	*N* = 8,560	*N* = 2,562		*N* = 11,500	*N* = 782	*N* = 10,781	*N* = 2,189	
Age (years)	39.3(0.2)	50.5(0.8)	40.6(0.2)	46.2(0.5)	<0.001	38.2(0.2)	45.1(1.2)	42.3(0.2)	48.0(0.5)	<0.001	37.4(0.2)	46.6(0.9)	42.4(0.2)	48.0(0.5)	<0.001
Sex (%)					<0.001					<0.001					<0.001
Male	50.8(0.4)	56.9(1.9)	46.7(0.7)	50.8(1.5)		55.6(0.5)	75.6(2.7)	39.7(0.6)	50.4(1.3)		59.4(0.5)	71.1(2.3)	38.9(0.6)	47.9(1.4)	
Female	49.2(0.4)	43.1(1.9)	53.3(0.7)	49.2(1.5)		44.4(0.5)	24.4(2.7)	60.3(0.6)	49.6(1.3)		40.6(0.5)	28.9(2.3)	61.1(0.6)	52.1(1.4)	
Ethnicity (%)					<0.001					<0.001					<0.001
Black	9.2(0.6)	1.0(0.2)	15.6(1.0)	3.5(0.5)		10.9(0.7)	0.7(0.3)	11.6(0.8)	2.8(0.3)		10.0(0.6)	1.0(0.3)	12.5(0.8)	3.1(0.4)	
White	69.9(1.1)	54.1(2.7)	65.4(1.4)	63.7(2.0)		67.8(1.2)	29.2(3.8)	69.7(1.3)	63.8(1.9)		67.5(1.1)	43.7(3.0)	69.7(1.3)	64.9(2.0)	
Mexican	7.4(0.5)	20.3 (1.9)	8.7(0.7)	19.4(1.6)		7.8(0.5)	34.1(3.2)	7.8(0.7)	18.1(1.6)		7.8(0.5)	24.2(2.2)	7.8(0.6)	18.5(1.6)	
Other	13.4(0.7)	24.6(2.2)	10.4(0.7)	13.5(1.2)		13.4(0.7)	35.9(3.4)	11.0(0.8)	15.3(1.2)		14.7(0.8)	31.2(2.6)	10.1(0.7)	13.6(1.2)	
Education level (%)					<0.001					<0.001					<0.001
Less than high school	15.2(0.6)	36.3(1.9)	15.5(0.6)	26.7(1.4)		15.3(0.6)	47.3(3.1)	15.1(0.6)	28.2(1.2)		14.8(0.6)	36.0(2.2)	15.7(0.6)	28.5(1.3)	
High school graduates	22.9(0.6)	24.7(1.6)	25.4(0.7)	29.8(1.6)		22.4(0.6)	23.6(2.6)	25.7(0.6)	28.5(1.3)		22.3(0.7)	26.0(1.8)	25.1(0.6)	28.5(1.4)	
Above high school	62.0(1.0)	38.9(2.0)	59.1(0.8)	43.6(1.9)		62.3(1.0)	29.1(2.8)	59.2(0.9)	43.4(1.5)		62.9(1.0)	38.1(2.1)	59.2(0.8)	43.0(1.6)	
Marital status (%)					0.340					0.003					<0.001
Separated	36.3(0.7)	34.8(1.7)	34.9(0.9)	34.0(1.8)		37.0(0.8)	34.5(2.9)	34.0(0.8)	34.3(1.4)		37.9(0.8)	33.0(2.3)	33.7(0.7)	34.7(1.6)	
Married	63.8(0.7)	65.2(1.7)	65.1(0.9)	66.0(1.8)		63.0(0.8)	65.5(2.9)	66.0(0.8)	65.7(1.4)		62.1(0.8)	67.1(2.3)	66.4(0.7)	65.3(1.6)	
PIR (%)					<0.001					<0.001					<0.001
<1.3	19.9(0.8)	30.8(2.2)	21.3(0.7)	28.6(1.6)		20.5(0.7)	40.1(3.1)	20.2(0.8)	28.2(1.6)		20.1(0.8)	34.0(2.4)	20.7(0.7)	28.1(1.6)	
1.3–3.5	33.8(0.8)	42.2(2.2)	37.4(0.9)	41.7(2.0)		34.1(0.7)	39.1(3.5)	36.2(0.8)	42.2(1.7)		33.5(0.8)	39.0(2.6)	36.4(0.8)	42.7(1.9)	
≥3.5	46.3(1.1)	27.0(2.2)	41.3(1.1)	29.7(1.7)		45.4(1.1)	20.9(3.4)	43.7(1.0)	29.6(1.6)		46.4(1.1)	27.0(2.3)	43.0(1.1)	29.2(1.6)	
Smoking status (%)					<0.001					<0.001					<0.001
Never	53.3(0.7)	52.0(1.9)	54.5(0.8)	53.4(1.6)		53.2(0.8)	54.3(3.3)	54.4(0.7)	52.8(1.3)		54.1(0.8)	50.7(2.7)	53.1(0.7)	53.6(1.4)	
Former	21.3(0.5)	29.5(1.7)	23.8(0.7)	28.3(1.3)		19.9(0.5)	27.3(2.8)	25.5(0.7)	28.9(1.1)		19.6(0.6)	28.3(2.3)	24.7(0.6)	28.8(1.2)	
Current	25.4(0.7)	18.4(1.5)	21.8(0.7)	18.3(1.4)		27.0(0.8)	18.4(2.4)	20.0(0.5)	18.4(1.0)		26.3(0.7)	21.0(1.9)	22.2(0.5)	17.6(1.1)	
Alcohol consumption (%)					<0.001					<0.001					<0.001
None	9.9(0.6)	21.4(1.6)	11.3(0.8)	14.7(1.2)		9.0(0.6)	16.5(2.0)	12.4(0.8)	17.1(0.9)		9.0(0.7)	17.5(1.8)	11.8(0.7)	16.9(1.1)	
Mild	35.6(0.8)	30.6(1.9)	32.2(0.9)	29.6(1.7)		35.0(0.7)	32.8(3.2)	33.9(0.9)	29.6(1.5)		36.8(0.8)	31.8(2.6)	32.2(0.8)	29.5(1.5)	
Moderate	19.0(0.5)	7.7(1.0)	17.5(0.7)	12.9(1.2)		18.9(0.5)	6.5(1.6)	18.0(0.6)	11.6(0.9)		18.7(0.6)	9.1(1.3)	18.4(0.6)	11.6(1.0)	
Heavy	24.3(0.6)	17.0(1.4)	23.7(0.8)	20.2(1.6)		26.8(0.7)	22.8(2.5)	19.9(0.7)	18.7(1.2)		25.9(0.7)	21.2(1.8)	22.3(0.6)	18.5(1.3)	
Former	11.2(0.5)	23.3(1.5)	15.2(0.8)	22.7(1.4)		10.3(0.4)	21.4(3.1)	15.8(0.8)	23.1(1.2)		9.8(0.5)	20.5(2.0)	15.3(0.7)	23.5(1.2)	
Physical activity (%)					<0.001					<0.001					<0.001
Never	39.2(0.8)	58.0(1.8)	42.4(0.7)	53.8(1.8)		38.1(0.8)	56.0(3.5)	43.6(0.7)	55.2(1.4)		37.5(0.8)	57.3(2.6)	43.1(0.7)	54.7(1.5)	
Moderate	26.3(0.5)	26.2(1.6)	28.2(0.7)	26.2(1.7)		25.1(0.6)	20.5(2.7)	29.8(0.6)	26.9(1.2)		24.4(0.6)	21.3(2.2)	29.5(0.6)	27.6(1.3)	
Vigorous	34.5(0.7)	15.9(1.4)	29.4(0.7)	20.0(1.6)		36.9(0.8)	23.5(2.9)	26.6(0.7)	17.9(1.3)		38.1(0.8)	21.5(2.0)	27.4(0.6)	17.7(1.4)	
BUN (mg/dL)	12.7(0.1)	13.6(0.2)	12.7(0.1)	12.9(0.2)	0.005	12.7(0.1)	13.9(0.4)	12.6(0.1)	13.0(0.2)	0.002	12.7(0.1)	13.5(0.3)	12.7(0.1)	13.0(0.2)	0.009
Scr (mg/dL)	0.9(0.0)	0.8(0.0)	0.9(0.0)	0.8(0.0)	<0.001	0.9(0.0)	0.8(0.0)	0.8(0.0)	0.8(0.0)	<0.001	0.9(0.0)	0.8(0.0)	0.8(0.0)	0.8(0.0)	<0.001
Protein intake (gm)	87.1(0.6)	73.5(1.8)	87.5(0.7)	77.8(1.5)	<0.001	90.0(0.6)	84.3(2.8)	82.4(0.7)	75.4(1.3)	<0.001	89.9(0.6)	79.1(1.9)	84.1(0.6)	75.6(1.4)	<0.001
Energy intake (kcal/day)	2319.6 (11.4)	1836.9 (35.5)	2303.8 (16.5)	2056.6 (34.7)	<0.001	2396.2 (12.5)	2038.7 (63.8)	2175.2 (15.7)	1977.9 (26.9)	<0.001	2393.6 (12.7)	1956.3 (42.9)	2224.2 (14.0)	1992.4 (29.5)	<0.001
Hypertension (%)	24.7(0.6)	48.8(2.1)	43.3(0.8)	55.7(1.9)	<0.001	23.4(0.5)	40.7(3.3)	41.6(0.8)	54.7(1.5)	<0.001	20.0(0.5)	39.8(2.4)	41.6(0.7)	57.0(1.6)	<0.001
Diabetes (%)	5.0(0.2)	16.8(1.3)	14.6(0.5)	26.8(1.4)	<0.001	5.4(0.3)	18.8(2.4)	11.9(0.4)	23.7(1.2)	<0.001	3.5(0.2)	11.3(1.3)	12.6(0.4)	26.6(1.4)	<0.001
Hyperlipidemia (%)	61.6(0.6)	79.9(1.6)	79.8(0.6)	82.8(1.4)	<0.001	59.7(0.6)	74.6(2.9)	79.2(0.6)	82.6(1.2)	<0.001	55.9(0.6)	76.2(2.3)	79.3(0.6)	83.4(1.2)	<0.001

LLM, low lean mass; BMI, body mass index; BF%, body fat percentage; WC, waist circumference; PIR, poverty income ratio; BUN, blood urea nitrogen; SCr, serum creatinine.

**Figure 2:**
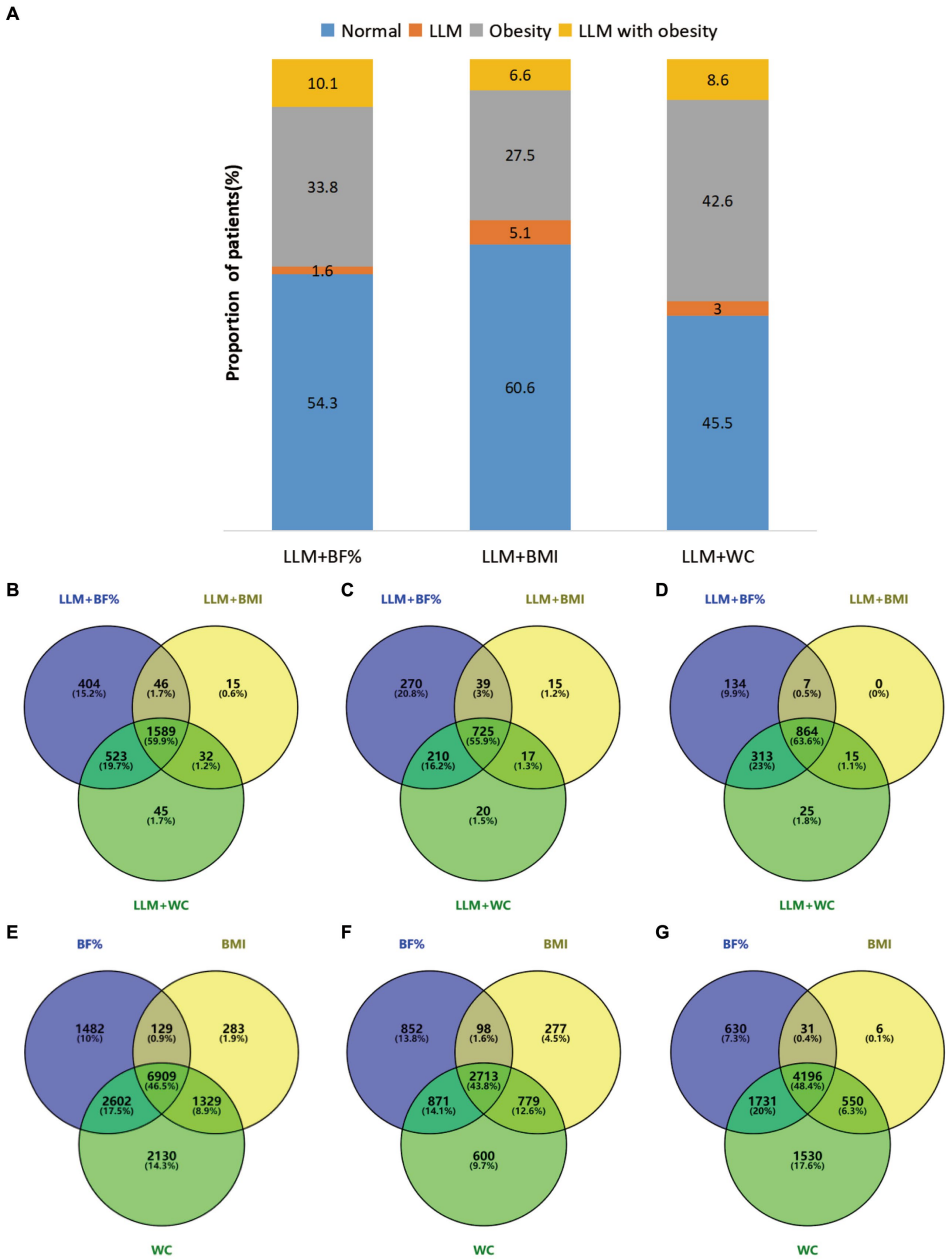
(A) Presented the prevalence of normal, LLM, obesity, and LLM with obesity, as determined by different obesity definitions. **(B–G)** Employed Venn diagrams to illustrate the agreement among various diagnostic criteria in the total population and different genders. Specifically **(B–D)**, respectively, examined the agreement of LLM with obesity in the overall population, males, and females, while **(E–G)** compared the agreement of obesity across three distinct obesity definitions in the overall population, males, and females. Diagram numbers show the number of participants. LLM, low lean mass; BMI, body mass index; BF%, body fat percentage; WC, waist circumference.

Figure 2 depicts the prevalence of normal, LLM, obesity, and LLM accompanied by obesity according to the different obesity diagnostic methods. Considering obesity alone, the incidence rate was relatively high when WC was used (42.6%), followed by BF% (33.8%) and BMI (27.5%). The incidence rate of LLM alone was comparatively low, with fluctuations ranging from 1.6 to 5.1%. Approximately half of the participants did not exhibit either LLM or obesity, accounting for 54.3% for LLM + BF%, 60.6 for LLM + BMI, and 45.5% for LLM + WC, respectively. The highest prevalence of LLM accompanied by obesity was observed when LLM was combined with BF% (10.1%), followed by LLM combined with WC (8.6%), and LLM combined with BMI (6.6%). The Venn diagrams illustrated in Figures 2B–D show that 59.9, 55.9, and 63.6% of participants in the total population, males, and females, respectively, satisfied all three diagnostic criteria for LLM accompanied by obesity. Additionally, Figures 2E–G demonstrate that 46.5, 43.8, and 48.4% of participants in the overall population, males, and females, respectively, met all three diagnostic criteria for obesity.

Overall, in Cohen’s kappa analysis of Table 2, the diagnostic agreement of obesity, as determined by BF%, BMI, and WC, was found to be moderate. LLM accompanied by obesity, defined by LLM plus BMI, LLM plus BF%, and LLM plus WC, indicated a relatively good agreement. Notably, the combination of LLM and WC demonstrated the best agreement, with κ values of 0.660 for LLM combined with BMI and 0.665 for LLM combined with BF%. When subgrouped by gender, LLM plus WC exhibited strong agreement with LLM plus BMI in males (*κ* =0.739). Strong agreement was observed among the three diagnostic methods in subjects aged 20–40 (all *κ* > 0.60). However, low agreement was noted in individuals over 60 years of age when using BMI for obesity diagnosis.

**Table 2:** Agreement between different diagnostic methods of LLM with obesity.

		Cohen’s kappa					
		Total	Male	Female	20 ≤ Age < 40	40 ≤ Age < 60	Age ≥ 60
BMI	BF%	0.531	0.507	0.541	0.611	0.531	0.384
BMI	WC	0.596	0.682	0.507	0.693	0.589	0.418
BF%	WC	0.600	0.609	0.558	0.623	0.564	0.544
LLM + BMI	LLM + BF%	0.604	0.592	0.608	0.646	0.598	0.527
LLM + BMI	LLM + WC	0.660	0.739	0.580	0.722	0.649	0.560
LLM + BF%	LLM + WC	0.665	0.673	0.637	0.657	0.632	0.688

LLM, low lean mass; BMI, body mass index; BF%, body fat percentage; WC, waist circumference.

The relationship between the risk of hyperuricemia and adverse body compositions was analyzed through weighted multivariate logistic regression analyses, as presented in Table 3. In the fully adjusted model, individuals with LLM exhibited a positive association with an elevated likelihood of hyperuricemia in comparison to those without LLM (OR: 1.49, 95% CI: 1.26–1.76). Likewise, upon accounting for all confounding variables, participants classified as obese based on BF%, WC, and BMI demonstrated significantly elevated odds of hyperuricemia than individuals without obesity (OR: 2.51, 95% CI: 2.25–2.80 for BMI; OR: 2.26, 95% CI: 2.03–2.52 for BF%; OR: 2.55, 95% CI: 2.25–2.88 for WC). In comparison to the individual without LLM and obesity, the adjusted risk of hyperuricemia exhibited a significant increase in LLM accompanied by obese individuals when defining obesity using BMI, BF%, and WC, with respective increments of 2.70 (2.23, 3.28), 2.44 (2.06, 2.89), and 3.12 (2.58, 3.76) times. Individuals with obesity alone exhibited significantly higher adjusted ORs for hyperuricemia compared to the normal group, as determined by LLM + BMI, LLM + BF%, and LLM + WC classifications. The risk of developing hyperuricemia was remarkably elevated in individuals with LLM alone, as defined by WC and BMI, compared to the healthy population. Nevertheless, this increased risk was not observed when LLM alone was defined by BF% in the fully adjusted models.

**Table 3:** Associations of adverse body compositions with hyperuricemia.

	Model I		Model II		Model III	
	OR (95% CI)	*P*	OR (95% CI)	*P*	OR (95% CI)	*P*
LLM	1.67 (1.48, 1.89)	<0.001	1.60 (1.41, 1.81)	<0.001	1.49 (1.26, 1.76)	<0.001
BMI	2.93 (2.69, 3.19)	<0.001	2.87 (2.60, 3.16)	<0.001	2.51 (2.25, 2.80)	<0.001
BF%	2.54 (2.33, 2.77)	<0.001	2.54 (2.31, 2.78)	<0.001	2.26 (2.03, 2.52)	<0.001
WC	3.01 (2.71, 3.34)	<0.001	2.99 (2.69, 3.32)	<0.001	2.55 (2.25, 2.88)	<0.001
LLM + BMI						
Normal	Reference		Reference		Reference	
LLM	1.56 (1.29, 1.89)	<0.001	1.52 (1.25, 1.86)	<0.001	1.41 (1.13, 1.76)	0.003
Obesity	2.98 (2.70, 3.29)	<0.001	2.93 (2.63, 3.26)	<0.001	2.54 (2.25, 2.87)	<0.001
LLM with obesity	3.12 (2.69, 3.61)	<0.001	2.98 (2.55, 3.48)	<0.001	2.70 (2.23, 3.28)	<0.001
LLM + BF%						
Normal	Reference		Reference		Reference	
LLM	1.18 (0.80, 1.75)	0.400	1.14 (0.74, 1.78)	0.540	1.26 (0.80, 2.01)	0.310
Obesity	2.50 (2.28, 2.74)	<0.001	2.51 (2.27, 2.77)	<0.001	2.24 (2.01, 2.51)	<0.001
LLM with obesity	2.80 (2.47, 3.18)	<0.001	2.72 (2.39, 3.10)	<0.001	2.44 (2.06, 2.89)	<0.001
LLM + WC						
Normal	Reference		Reference		Reference	
LLM	1.53 (1.21, 1.94)	<0.001	1.46 (1.12, 1.90)	0.005	1.44 (1.06, 1.96)	0.020
Obesity	2.98 (2.66, 3.34)	<0.001	2.97 (2.64, 3.33)	<0.001	2.53 (2.21, 2.90)	<0.001
LLM with obesity	3.74 (3.21, 4.37)	<0.001	3.62 (3.11, 4.22)	<0.001	3.12 (2.58, 3.76)	<0.001

OR, odds ratio; 95% CI, 95% confidence interval; LLM, low lean mass; BMI, body mass index; BF%, body fat percentage; WC, waist circumference. Model I adjusted for age, sex, and ethnicity. Model II adjusted for model I plus education level, marital status, PIR, smoking status, alcohol consumption, and physical activity. Model III adjusted for model II plus BUN, Scr, protein intake, energy intake, hypertension, diabetes, and hyperlipidemia.

The stratified analyses showed that individuals with both LLM and obesity had a greater likelihood of developing hyperuricemia compared to healthy individuals, even when considering various age and gender subgroups (Table 4). This association was particularly pronounced among females in total population even when employing three different definitions of obesity (all *P*_interaction_ < 0.001). Moreover, the positive relationship between LLM accompanied by obesity and hyperuricemia was stronger among participants aged 20–40 when BF% was applied to classify obesity (*P*_interaction_ = 0.040). Given the likelihood of variations in this association among males and females across different age groups, we further performed subgroup analyses on the youth, middle-aged, and elderly populations of both genders, as depicted in Table 5. Among participants aged 20–40, males exhibited a more pronounced correlation between LLM accompanied by obesity and hyperuricemia compared to females when utilizing BMI and WC as indicators of obesity (both *P*_interaction_ < 0.05). Conversely, among participants aged 40–60, this relationship was more prominent in females when using three distinct definitions of obesity (all *P*_interaction_ < 0.05).

**Table 4:** Subgroup analysis for the association between adverse body compositions with hyperuricemia based on sex and age.

		Normal	LLM	Obesity	LLM with Obesity	*P* for interaction
		OR (95% CI)	OR (95% CI)	OR (95% CI)	OR (95% CI)	
LLM + BMI	Sex					<0.001
	Male	Reference	1.39 (1.04, 1.87)	2.14 (1.85, 2.46)	2.46 (1.90, 3.20)	
	Female	Reference	1.77 (1.15, 2.72)	3.46 (2.87, 4.17)	3.56 (2.58, 4.91)	
	Age					0.187
	20 ≤ age < 40	Reference	2.18 (1.23, 3.88)	2.91 (2.44, 3.47)	3.82 (2.65, 5.52)	
	40 ≤ age < 60	Reference	1.42 (0.89, 2.29)	2.37 (1.96, 2.87)	2.45 (1.88, 3.19)	
	≥60	Reference	1.18 (0.82, 1.70)	2.46 (1.88, 3.22)	2.32 (1.70, 3.15)	
LLM + BF%	Sex					<0.001
	Male	Reference	0.90 (0.54, 1.48)	2.09 (1.82, 2.39)	2.36 (1.89, 2.93)	
	Female	Reference	5.35 (2.20, 13.01)	2.56 (2.12, 3.09)	2.92 (2.13, 4.00)	
	Age					0.040
	20 ≤ age < 40	Reference	1.41 (0.66, 2.98)	2.87 (2.45, 3.37)	3.67 (2.59, 5.20)	
	40 ≤ age < 60	Reference	1.25 (0.59, 2.66)	1.90 (1.57, 2.30)	2.13 (1.69, 2.68)	
	≥60	Reference	1.24 (0.54, 2.88)	2.03 (1.53, 2.68)	2.02 (1.50, 2.71)	
LLM + WC	Sex					<0.001
	Male	Reference	1.34 (0.95,1.90)	2.32 (1.20, 2.71)	2.89 (2.24, 3.72)	
	Female	Reference	3.19 (1.68, 6.05)	3.67 (2.85, 4.72)	4.76 (3.30, 6.86)	
	Age					0.295
	20 ≤ age < 40	Reference	1.65 (0.82, 3.34)	3.10 (2.55, 3.78)	4.62 (3.16, 6.75)	
	40 ≤ age < 60	Reference	1.53 (0.95, 2.47)	2.26 (1.81, 2.81)	2.72 (2.13, 3.49)	
	≥60	Reference	1.48 (0.96, 2.30)	2.55 (1.89, 3.43)	2.72 (1.92, 3.89)	

Abbreviations as in Table 3.

**Table 5:** Subgroup analysis for the association between adverse body compositions with hyperuricemia based on youth, middle-aged, and elderly populations in males and females.

		Normal	LLM	Obesity	LLM with Obesity	*P* for interaction
		OR (95% CI)	OR (95% CI)	OR (95% CI)	OR (95% CI)	
LLM + BMI	20 ≤ age < 40					0.004
	Male	Reference	1.90 (1.01, 3.57)	2.46 (1.97, 3.08)	4.44 (2.81, 7.03)	
	Female	Reference	2.85 (0.98, 8.28)	4.29 (3.12, 5.94)	3.59 (1.74, 7.42)	
	40 ≤ age < 60					< 0.001
	Male	Reference	1.05 (0.58, 1.90)	1.95 (1.54, 2.47)	2.09 (1.36, 3.21)	
	Female	Reference	2.64 (1.23, 5.67)	3.10 (2.33, 4.14)	3.31 (2.05, 5.34)	
	≥60					0.007
	Male	Reference	1.14 (0.74, 1.77)	1.83 (1.19, 2.83)	1.44 (0.93, 2.23)	
	Female	Reference	1.33 (0.71, 2.52)	3.18 (2.25, 4.49)	3.71 (2.34, 5.89)	
LLM + BF%	20 ≤ age < 40					0.245
	Male	Reference	1.09 (0.44, 2.70)	2.66 (2.14, 3.29)	4.05 (2.75, 5.96)	
	Female	Reference	4.07 (1.20, 13.88)	3.25 (2.36, 4.49)	3.10 (1.53, 6.28)	
	40 ≤ age < 60					0.003
	Male	Reference	0.69 (0.29, 1.62)	1.72 (1.35, 2.20)	1.90 (1.31, 2.76)	
	Female	Reference	8.32 (1.81, 38.22)	2.27 (1.63, 3.15)	2.75 (1.69, 4.47)	
	≥60					0.178
	Male	Reference	0.85 (0.31, 2.35)	2.03 (1.39, 2.98)	1.70 (1.16, 2.49)	
	Female	Reference	2.82 (0.64, 12.39)	2.18 (1.31, 3.61)	2.68 (1.65, 4.36)	
LLM + WC	20 ≤ age < 40					0.017
	Male	Reference	1.39 (0.66, 2.92)	2.76 (2.22, 3.44)	5.41 (3.44, 8.50)	
	Female	Reference	4.93 (1.24, 19.71)	4.27 (2.85, 6.38)	4.45(2.11, 9.41)	
	40 ≤ age < 60					< 0.001
	Male	Reference	1.39 (0.82, 2.37)	2.01 (1.55, 2.60)	2.20 (1.47, 3.29)	
	Female	Reference	3.08 (1.05, 9.03)	3.70 (2.31, 5.93)	5.26 (3.04, 9.10)	
	≥60					0.152
	Male	Reference	1.20 (0.70, 2.05)	2.28 (1.59, 3.26)	2.05 (1.33, 3.16)	
	Female	Reference	2.76 (1.00, 7.89)	3.09 (1.74, 5.49)	3.99 (2.02, 7.87)	

Abbreviations as in Table 3.

## Discussion

This analysis is the first to compare the agreement of LLM accompanied by obesity and examine its correlation with hyperuricemia. The results indicated that the overall diagnostic agreement of LLM with obesity was moderate to strong. Specifically, the combination of LLM and WC exhibited the highest level of agreement with other criteria in the overall population, as well as among males and patients of different age groups. LLM accompanied by obesity was linked to an elevated risk of hyperuricemia, even when three different diagnostic methods were used, thereby supporting our initial hypothesis. Moreover, this association remained prominent and consistent across the various subgroups categorized by age and sex. Notably, this relationship exhibited a marked disparity between genders, with males aged 20–40 showing a particularly strong association, while females aged 40–60 displayed a more pronounced relationship.

Varying definitions of obesity may result in significant differences in the approaches used to measure and diagnose SO. While BMI has traditionally served as an indicator of obesity, its susceptibility to misdiagnosis or missed diagnosis is evident, especially among older individuals, as it fails to differentiate between lean mass and fat mass (29). Our findings also indicated poor agreement among participants aged above 60 when defining obesity based on BMI. Consequently, alternative indicators, such as WC, BF%, visceral fat area (VFA), and waist-to-hip ratio, have been proposed for evaluating obesity. Considering previous studies on SO and the availability of the NHANES database, we selected BF%, WC, and BMI as indicators for diagnosing obesity (14, 20, 21). The incidence rate of LLM with obesity fluctuated between 6.6 and 10.1% in this analysis, rendering comparisons with other studies challenging because most studies have focused on the elderly population. A meta-analysis reported that the global prevalence of SO among the elderly population was about 11%, with a notably higher prevalence of approximately 20% observed among the American population (30). In another research, where ALM/BMI was combined with BF% in the older population of the United States, it was indicated that the occurrence of SO was 12.6% among males and 33.5% among females (31). This can be explained because aging is commonly accompanied by a decline in muscle mass and an increase in visceral adipose tissue. Our study indicated that there was moderate to strong diagnostic agreement between LLM accompanied by obesity across various demographic groups, including the overall population, males, females, youth, and middle-aged individuals. This suggests that applying three different methods may effectively identify participants with LLM accompanied by obesity. In addition, our results presented that the combination of LLM and WC had the best agreement with other criteria in the overall population, males, and patients of different age groups. This observation aligns with prior investigations (21, 32), which suggested that WC serves as a reliable diagnostic tool for identifying individuals with SO. This is not unexpected because WC can indirectly assess the accumulation of visceral fat, whereas BMI and BF% cannot reflect regional adiposity.

In agreement with prior studies, we observed a significant correlation between obesity, as determined by BMI, BF%, and WC, and an increased likelihood of hyperuricemia. Previous investigations have established a connection between the risk of hyperuricemia and indicators of obesity, including VFA, BMI, WC, and WWI (16–18, 33). Moreover, our findings suggested a significant association between LLM and an increased susceptibility to hyperuricemia. This findings aligns with research implemented by Zhou et al. (34), which identified a negative relationship between sarcopenia and uric acid levels in middle-aged and older Chinese individuals. Similarly, two other cross-sectional studies also demonstrated an association between hyperuricemia and reduced skeletal muscle mass (35) and muscle strength (36). However, the relationship between LLM accompanied by obesity and hyperuricemia remains unclear. The loss of muscle mass accompanied by obesity is commonly regarded as an age-related condition, leading most studies to focus solely on the elderly. Nevertheless, it is crucial to implement early interventions, starting at a young age. Our findings suggest that LLM accompanied by obesity was linked to an elevated risk of hyperuricemia, whether in youth, middle-aged, or elderly populations. This association appeared to be particularly noteworthy in young individuals (20–40), as the adjusted ORs for hyperuricemia were higher than in individuals aged above 40 when obesity was defined as BF%. Interestingly, we observed that the association between LLM accompanied by obesity and hyperuricemia was particularly pronounced in males when participants aged 20–40, while this association was more significant in females when participants aged 40–60. Additionally, similar to previous studies (33, 37), we found a remarkably lower prevalence of hyperuricemia in females than in male. This gender difference may be associated with sex hormone. It is plausible that estrogen enhances uric acid excretion by inhibiting the expression of urate reabsorptive transporters at the protein level (38, 39). Besides, animal studies suggested that the secretion of male-pattern growth hormone led to the down-regulation of the expression of the urate transporter glucose transporter 9, whose inactivation resulted in hyperuricemia (40, 41). Accordingly, it may be important to consider gender disparities in hyperuricemia prevention among individuals with LLM accompanied by obesity.

Several potential mechanisms have been suggested to elucidate the correlation between SO and hyperuricemia. On the one hand, insulin resistance (IR) plays a pivotal role between SO and hyperuricemia. Skeletal muscle comprises 40–50% of lean body mass in adult humans and serves as the primary insulin-sensitive organ. Sarcopenia can trigger various pathways leading to IR (42, 43). Obesity can induce IR through macrophage infiltration and low-grade inflammation (44). Thus, impaired insulin signaling is frequently observed in individuals with SO, which contributes to reduced uric acid excretion and increased synthesis (45, 46). In turn, uric acid can induce IR via activation of the NOD-like receptor family pyrin domain containing three inflammasomes (47). Furthermore, IR promotes skeletal muscle reduction by increasing the amount of myostatin and aggravating obesity by inhibiting the decomposition of visceral fat (48, 49). On the other hand, it has been discovered that leptin, a factor derived from adipose tissue, can promote the synthesis of inflammatory cytokines, thus playing a role in the development of sarcopenia (43). Moreover, researches has shown a positive link between higher levels of leptin and an increased in uric acid levels (50, 51). Furthermore, the presence of uric acid can stimulate the release of certain inflammatory substances (52), which may drive sarcopenia and obesity (53). Hyperuricemia is a metabolic disorder and its specific interaction mechanism with SO has not yet been clear. Nonetheless, a close relationship between SO and metabolic disease has been established. Consequently, evaluating LLM accompanied by obesity is important in predicting the risk of hyperuricemia. Our study suggests a strong association between LLM with obesity and an increased risk of hyperuricemia.

This study demonstrated notable strengths, including the incorporation of a substantial sample size and the utilization of strong statistical methods to assess the agreement of LLM accompanied by obesity, as well as to investigate its association with hyperuricemia by employing different definitions of obesity. Nevertheless, it is crucial to recognize the intrinsic limitations of this analysis. First, LLM was defined solely based on low muscle mass and did not encompass low muscle strength and physical performance, as only the 2011–2012 and 2013–2014 cycles had available data, which may inadequately capture the correlation between sarcopenic obesity and hyperuricemia. Consequently, the term “sarcopenia” was not employed; instead, “LLM” was utilized to describe low muscle mass. The definition of low muscle mass employed in this investigation has been acknowledged and implemented in recent studies (31, 54). Second, there are other approaches for defining obesity, such as visceral fat area and android/gynoid fat mass. Third, we only used the FNIH criterion to define LLM, and it is unclear whether the results are consistent when employing different LLM criteria. Lastly, the cross-sectional study only describes the association between LLM accompanied by obesity and hyperuricemia and cannot be expected to elucidate the pathomechanisms.

## Conclusion

When the three obesity indicators were employed to define LLM with obesity, the diagnostic agreement was relatively good. Moreover, we found a strong relationship between LLM accompanied by obesity and an increased risk of hyperuricemia. Timely intervention in individuals with LLM and obesity may potentially reduce hyperuricemia. Further research is warranted, particularly in prospective studies with larger sample sizes that specifically consider the influence of gender and age.

## Data availability statement

Publicly available datasets were analyzed in this study. This data can be found here: All data are publicly available and can be accessed at the NHANES (https://www.cdc.gov/nchs/nhanes/index.htm).

## Ethics statement

The study was performed in accordance with the Declaration of Helsinki, and the survey protocol was approved by the Research Ethics Review Board of the NCHS (https://www.cdc.gov/nchs/nhanes/irba98.htm).

## Author contributions

HX: Formal analysis, Methodology, Software, Visualization, Writing – original draft. LH: Data curation, Resources, Writing – original draft. MX: Data curation, Resources, Writing – original draft. YD: Data curation, Writing – original draft. DL: Supervision, Writing – review & editing.
